# Social Memory and the Role of the Hippocampal CA2 Region

**DOI:** 10.3389/fnbeh.2019.00233

**Published:** 2019-10-01

**Authors:** Nikolaos Tzakis, Matthew R. Holahan

**Affiliations:** Department of Neuroscience, Carleton University, Ottawa, ON, Canada

**Keywords:** CA2, social memory, hippocampus, social recognition, social neuroscience

## Abstract

The CA2 region of the hippocampus is a somewhat obscure area lacking in an understanding of its form and function. Until recently, the CA2 has been thought of as merely an extension of the CA3, with some referring to it as the CA3a region. Recent investigations into this area have not only delineated the CA2, but also defined it as a region distinct from both CA1 and CA3, with a unique set of cortical inputs and outputs and contributions to cognitive processes. One such process that has been shown to depend on the CA2 is the ability to recognize a novel or familiar conspecific, known as social recognition memory. Here, we review these findings and discuss the parallels between CA2 dysfunction and social impairments.

## Social Neuroscience

Social cognitive neuroscience is a burgeoning interdisciplinary field of research that aims to delineate the “why” and “how” of interactions between individuals. It aims to explore the relationship between cognitive processes and behavior in a social context. First described by Cacioppo and Berntson (1992), this field was developed to study mental and behavioral processes using an integrative, multimodal approach. This field of study is comprised of three levels of interaction: the social level, which focuses on the motivational and social factors that influence behavior and experience; the cognitive level, which focuses on the information-processing mechanisms that contribute to social-level processes; and the neural level, which focuses on the role of various brain regions and their involvement in cognitive level processing (Ochsner and Lieberman, 2001).

## Social Memory

One of the processes that is paramount to the structure and stability of relationship networks that define societies is that of social memory (Kogan et al., 2000). Social memory reflects different cognitive and behavioral processes, such as the ability to recognize a familiar or novel conspecific, commonly referred to as social recognition, or the ability to learn from others, commonly referred to as social learning (van der Kooij and Sandi, 2012). For the purpose of this review, we will predominantly focus on the social recognition aspect of social memory.

The ability to recognize a familiar versus novel individual is the foundation on which social relationships are built. Memory for individual conspecifics is not only necessary to engage in meaningful relationships but is also required to express appropriate behavioral responses based on previous encounters (van der Kooij and Sandi, 2012; Jacobs et al., 2016). Social recognition memory in animals is essential for social hierarchy, mate and offspring recognition, territorial defense, interspecies recognition, and for the general establishment and maintenance of groups (Ferguson et al., 2002; Jacobs et al., 2016). Social recognition depends on various sensory cues in order to establish the identity of an individual. Social recognition in humans and primates relies on the use of visual and auditory cues, with the right fusiform gyrus being responsible for coding information pertaining to the physical characteristics of an individual. Patients with lesions in the right fusiform gyrus are unable to recognize faces, an ailment known as prosopagnosia, while leaving all other visual and memory processes intact (Landis et al., 1986; Ferguson et al., 2002). While auditory and visual information may have important influences on social recognition in humans and other primates, chemosensory cues in the form of olfactory or pheromonal signals are more relied upon by most other mammals to encode social information (Ferguson et al., 2002). Chemically induced anosmia or removal of the vomeronasal organ in mice results in a decrease in individual recognition, providing evidence to suggest that intact olfactory processing is required for social recognition processes (Matochik, 1988; Bluthe and Dantzer, 1993; Kogan et al., 2000).

While social recognition is a common process across a variety of mammals, there are species and sex differences which may influence how it is expressed (van der Kooij and Sandi, 2012). Studies comparing social memory in mice and rats reported that the memory is much more resilient in mice than it is in rats. Recognition in rats was reported to only last about 30–60 min following acquisition as opposed to mice where it was reported to last for days. The opposite is true when comparing other types of memories, such as spatial memory, between the two species (Engelmann and Landgraf, 1994; Letty et al., 1997; Kogan et al., 2000; van der Kooij and Sandi, 2012). Differences in regional processing may account for the variations reported in these species. Following a social preference task, c-Fos expression in both the main and accessory olfactory bulb was significantly upregulated in mice where modest c-Fos expression was only observed in the accessory olfactory bulb in rats. This suggests that a difference in olfaction may account for the species-specific differences in social recognition (Noack et al., 2010; van der Kooij and Sandi, 2012).

Sex-differences have also been reported to exist in social recognition processes. In a social recognition task, female rats were reported to spend less time investigating a conspecific juvenile compared to males, yet females displayed stronger remote social recognition than males (Markham and Juraska, 2007; van der Kooij and Sandi, 2012). Based on these results, it is possible that estrogen activity may be a contributing factor that influences this behavior. Indeed, female mice lacking α and β estrogen receptors demonstrated impaired social recognition, while estrogen replacement in ovariectomized female mice improved social recognition (Choleris et al., 2003; Tang et al., 2005; van der Kooij and Sandi, 2012). Administration of an estrogen α receptor agonist impaired social recognition, while administration of an estrogen β receptor agonist prolonged it. Together, these results provide evidence to explain the presence of more robust social memory processes in females, which partly relies on estrogen receptor activity (Clipperton et al., 2008; van der Kooij and Sandi, 2012).

## Hippocampus and Social Memory

Evidence suggests that the hippocampus is required for proper processing of social memories as data have shown that hippocampal lesions disrupt social recognition (Maaswinkel et al., 1996). Rats with hippocampal transections were introduced to a juvenile rat for 5 min then reintroduced 5 min later. On the following day, the rats were introduced to a novel juvenile rat and their behaviors were recorded (Maaswinkel et al., 1996). Lesioned rats spent less time engaged in social investigation than control rats and were unable to distinguish between a familiar and unfamiliar juvenile. While they were unable to determine the precise nature of the impairment in social memory, they postulated that the deficits resulted from a disruption in sequential behavior or conditional association formation (Maaswinkel et al., 1996).

Kogan et al. (2000) also provided evidence to suggest that the hippocampus plays a role in social memory. They induced hippocampal lesions using ibotenic acid in adult mice then exposed the mice to a social recognition task. For this task, the experimental mouse was introduced to a juvenile mouse for an initial interaction of 2 min. The experimental mouse was then exposed to either the same or a novel juvenile mouse following either a 30-s or a 30-min intertrial interval. Hippocampal-lesioned mice showed intact social memory when tested after a 30-s delay but impaired social memory when tested after a 30-min delay (Kogan et al., 2000). The authors concluded that immediate social recognition memory is not impaired by hippocampal lesions but that long-term social memory is dependent on hippocampal function. They further postulated that the role of the hippocampus in integrating complex stimuli, particularly those from the olfactory bulb, is crucial in the recognition process and may explain why intact hippocampal function is essential for social memory (Kogan et al., 2000).

## Functional Divisions of the Hippocampus

**FIGURE 1:**
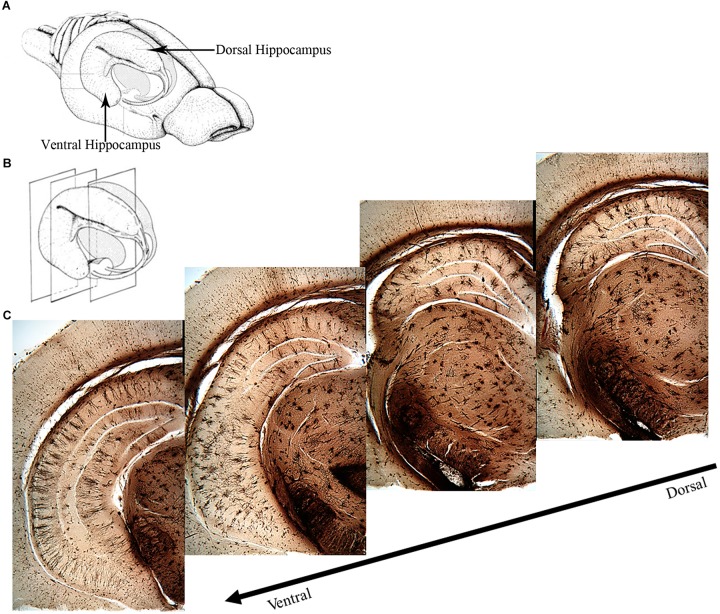
Overview of the rat hippocampus. **(A)** Drawing of the rat brain showing the three-dimensional organization of the hippocampus and locations of the dorsal and ventral hippocampus. **(B)** Coronal planes showing approximate locations of the images in **(C)**. **(C)** Golgi images illustrating the dorsal and ventral aspects of the hippocampus. The dorsal hippocampus is generally thought to mediate cognitive processes such as declarative memory formation and spatial learning and memory functions. Functional mapping studies have suggested that activity in the dorsal hippocampus relates to cortical regions that are involved in higher order information processing. The ventral hippocampus appears to be more involved with functions related to stress, emotion and affect. Neural activity in the ventral hippocampus corresponds to regions involved in emotion and stress (e.g., amygdala and hypothalamus). **(A,B)** Adapted from Cheung TH, Cardinal RN. Hippocampal lesions facilitate instrumental learning with delayed reinforcement but induce impulsive choice in rats. BMC Neurosci. 2005 May 13;6:36. 10.1186/1471-2202-6-36.; PMCID: PMC1156904. https://openi.nlm.nih.gov/detailedresult?img=PMC1156904_1471-2202-6-36-3&req=4. License information found here https://creativecommons.org/licenses/by/2.0/.

The two major divisions of the hippocampus are the dorsal and the ventral hippocampus (Figure 1), each with their own distinct input and output connections and roles in memory processing (Swanson and Cowan, 1977; Moser et al., 1995; Moser and Moser, 1998; Fanselow and Dong, 2010). The dorsal hippocampus (Figure 1) receives input from the caudolateral band of the entorhinal cortex and sends excitatory projections to dorsal parts of the subiculum, presubiculum, and post-subiculum. Additionally, the dorsal hippocampus sends projections to the retrosplenial and anterior cingulate cortices, as well as the caudal and rostral part of the lateral septal nucleus, an area that has been found to regulate social behavior (Fanselow and Dong, 2010; Bredewold et al., 2015). The ventral hippocampus (Figure 1) receives input from the rostromedial band of the entorhinal cortex and sends direct projections to the olfactory bulb, along with bi-directional projections to the amygdala (Saunders et al., 1988; Pitkanen et al., 2000; Cenquizca and Swanson, 2007; Fanselow and Dong, 2010).

The ventral hippocampus has been shown to be involved in emotional memory processes, like fear, and influences the fear memory processing of the amygdala, given that the ventral, but not the dorsal, hippocampus projects directly to the amygdala (Moser and Moser, 1998; Fanselow and Dong, 2010). The dorsal hippocampus contributes to spatial memory processes due to the observation that place fields in this region are more compacted whereas place fields in the ventral hippocampus are more dispersed (Jung et al., 1994). A study reported by Moser et al. (1993) supports this hypothesis. After performing localized dorsal hippocampal lesions and testing rats in the Morris water maze, they noted impaired latencies during training, along with a reduction in the amount of time spent in the target quadrant during the probe test. Rats with ventral hippocampus lesions experienced no impairment in their spatial learning abilities. Based on these results, they concluded that the dorsal hippocampus is more important for the encoding of new spatial information than the ventral aspect (Moser et al., 1993; Riedel and Micheau, 2001). Further evidence to support this hypothesis comes from studies completed by Moser et al. (1995) and Pothuizen et al. (2004), who also reported that hippocampal-dependent learning was impeded if the dorsal portion of the hippocampus was lesioned. They further noted that dorsal hippocampal lesions were just as effective as complete hippocampal lesions in disrupting spatial working memory, as opposed to lesions of the ventral hippocampus, which resulted in no disruption of spatial working memory.

## Subregions of the Dorsal Hippocampus

**FIGURE 2:**
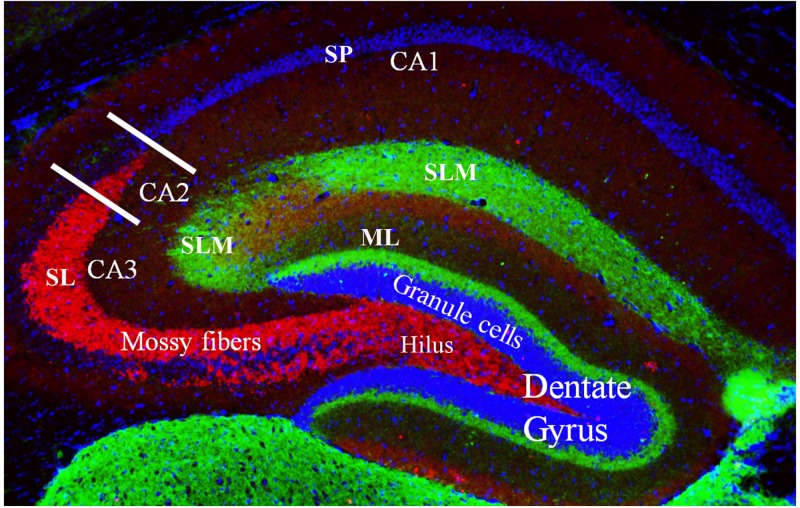
Coronal cross section of the dorsal hippocampus showing the primary subdivisions. The granule cells (stained with DAPI) make up the major cell type in the dentate gyrus. These neurons project to the CA3 and CA2 regions via the unmyelinated mossy fibers (shown in red, stained with a zinc transporter antibody). The mossy fibers have large presynaptic terminals that enable synaptic connections to be made with CA3 and CA2 pyramidal cells and the mossy cells of the polymorphic layer (or, hilus). Each mossy fiber gives rise to several thin collaterals that synapse in the CA3 *stratum lucidum* (SL) and CA2 *stratum radiatum* (SR). The CA areas are all filled with densely packed pyramidal cells that make up the *stratum pyramidale* (SP; only labeled in CA1 but layer also present in CA2 and CA3). Green staining shows axonal terminal fields in the *stratum lacunosum moleculare* (SLM) of the CA1, CA2, and CA3 subfields and the molecular layer (ML) of the dentate gyrus. These terminals mainly arise from the entorhinal cortex.

The dentate gyrus (Figure 2) receives multiple sensory inputs, including olfactory, auditory, and visual, from both the perirhinal and entorhinal cortices, along with spatially organized grid cells from the entorhinal cortex (Figure 2). These inputs allow the dentate gyrus to regulate, process, and represent both spatial and non-spatial information concurrently, commonly known as “conjunctive encoding” (Kesner, 2013).

The ability to organize and separate spatial events from one another, allowing an organism to temporally remember one place as distinct from another, is largely mediated by the dentate gyrus (Kesner, 2013). This spatial pattern separation is facilitated by mossy fibers (Figure 2) which form connections between granule cells in the dentate gyrus and pyramidal cells in the CA3 and CA2 regions and dictate which of these neurons will fire during learning based on activity in the dentate gyrus (O’Reilly and McClelland, 1994; Rolls, 1996; Kesner, 2013). Rats with dentate gyrus lesions were unable to discriminate object-place paired associates for reward; that is, they were impaired in their ability to distinguish between the same two objects placed in different locations (Lee and Solivan, 2010). Lesioned rats were able to discriminate between four different objects presented in the same location. Finally, using the same initial two objects, but placing them in remote locations, lesioned rats were initially impaired at discriminating the objects, but were able to relearn the task, showing no sustained deficits (Lee and Solivan, 2010). They concluded that the dentate gyrus is necessary for the ability to discriminate between object-place paired associates when object and/or spatial information overlaps but has less of an impact when that overlapping information decreases (Lee and Solivan, 2010). Other studies in the literature have corroborated these results suggesting that the deficits in spatial tasks resulting from dentate gyrus lesions may be a function of increased interference and impairment in spatial pattern separation (McDonald and White, 1995; Gilbert et al., 2001; Morris et al., 2012; Kesner, 2013).

**FIGURE 3:**
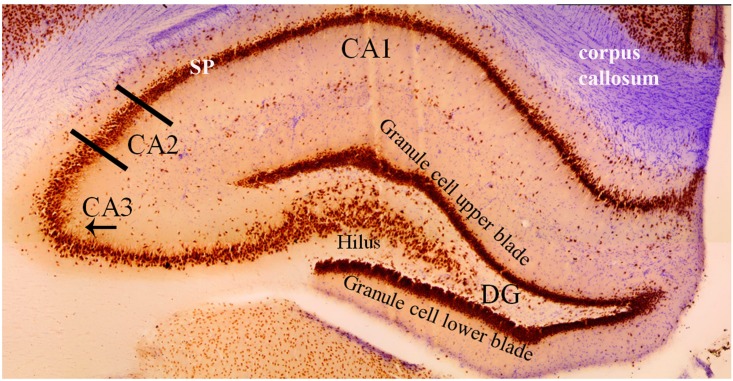
Coronal cross section of the dorsal hippocampus stained with the neuron specific antibody, NeuN. Image more clearly shows the various cell types in each hippocampal subfield. The NeuN protein is localized in the nuclei and perinuclear cytoplasm of most neurons in the central nervous system. The hippocampus proper is defined by the dentate gyrus and Cornu Ammonis (CA). The dentate gyrus contains densely packed granule cells in both an upper and lower blade. The hilus (also referred to as the polymorphic layer) within the granule cell layers contains mossy cells. In this coronal plane, the NeuN stain shows the base of the apical dendrite protruding from the CA3 pyramidal cells (arrow under “CA3”). This pattern of staining is absent from the CA2 pyramidal neurons indicating (1) the CA2 apical dendrites are not in the coronal plane and (2) an anatomical distinction between CA2 and CA3 pyramidal cells. Comparison of the NeuN staining in CA2 and CA1 regions shows the CA1 *stratum pyramidale* (SP) layer to be more densely packed and narrower than the CA2 region. Purple stain from a cresyl violet counterstain shows the corpus callosum.

The CA1, CA2, and CA3 regions are the principal pyramidal cell fields in the hippocampus (Figure 3) and are often the focus of research concerned with memory encoding and retrieval (McNaughton and Morris, 1987; Chevaleyre and Siegelbaum, 2010). The CA regions are each composed of layers, or “strata”: the *stratum pyramidale*, the *stratum lacunosum-moleculare*, the *stratum lucidum*, the *stratum oriens*, and the *stratum radiatum*. The *stratum pyramidale* contains the cell bodies of pyramidal cells and various interneurons (Andersen et al., 2007). Pyramidal cell layers in the CA1 are more tightly packed than those in the CA2 and CA3 regions (Figure 3).

**FIGURE 4:**
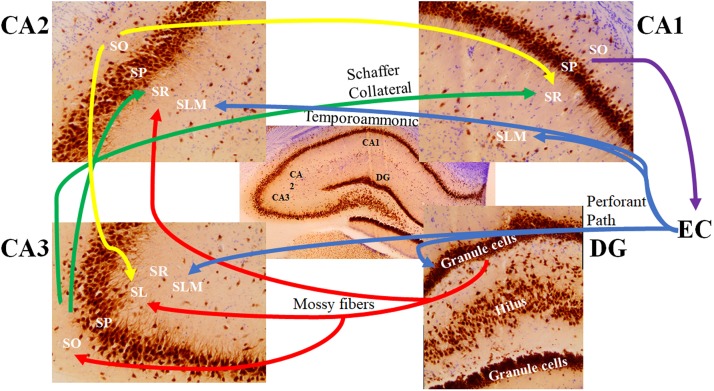
The major signaling pathways through the hippocampal subfields. The most prominent afferent input to the hippocampus originates from the entorhinal cortex (EC). The axons forming the perforant path (blue) arise from layer 2 of the EC and show synaptic contacts in the granule cell molecular layer of the dentate gyrus (DG) and the *stratum lacunosum moleculare* (SLM) of the CA3 region. There are also distinct projections from layer 3 of the EC to the SLM of the CA1 and CA2 regions (referred to as the temporoammonic pathway; blue). Granule cells of the DG send their axons (mossy fibers; red) through the hilus to the CA3 *stratum lucidum* (SL) and the border of the CA2 *stratum radiatum* (SR) and SLM. The mossy fibers also show terminal fields in the *stratum oriens* (SO) of the CA3 pyramidal cells. Pyramidal cells of the CA3 region send their axons (the Schaffer Collateral pathway; green) to the CA1 and CA2 SR subfields. Pyramidal cells of the CA2 project back to the CA3 SL region and forward to the CA1 SR subfield (yellow). Pyramidal cells of the CA1 region send their axons to the subiculum (not shown) and back to the deep layers of the EC (purple). SP: *stratum pyramidale.*

In the CA2 and CA3 regions, the *stratum lacunosum-moleculare* receives inputs from layers II and VI of the entorhinal cortex, while the *stratum lacunosum-moleculare* in the CA1 receives input from layers III and V of the entorhinal cortex (Witter and Amaral, 1991; van Groen et al., 2003; Figure 4). The *stratum lucidum*, which is found exclusively in the CA3 field between the pyramidal cell layer and the *stratum radiatum* (Figure 4), contains mossy fibers from the granule cells of the dentate gyrus (Witter and Amaral, 1991; Andersen et al., 2007). Axons from cells within the layer II of the entorhinal cortex synapse directly with the dendritic spines of granule cells in the dentate gyrus. Mossy fibers are formed by the axons of these granule cells and form synaptic connections with the proximal apical dendrites of pyramidal cells in the *stratum lucidum* of the CA3 via the hilus of the dentate fascia (Andersen et al., 1971; Sandler and Smith, 1991; Witter, 2007; Neves et al., 2008).

The *stratum oriens* is a deep, relatively cell-free layer containing the basal dendrites of the pyramidal cells and various interneurons, along with some of the myelinated Schaeffer collateral connections from CA3 to CA1 (Figure 4). The *stratum radiatum* possesses the majority of Schaeffer collateral connections (Andersen et al., 2007). Both the subiculum and the *stratum lacunosum-moleculare* of the CA1 also receive direct projections from neurons in layer III of the entorhinal cortex (Kajiwara et al., 2008; Maccaferri, 2011). Axons of CA1 pyramidal neurons project into the septum, hypothalamus, and contralateral hippocampus via the fimbria (Andersen et al., 1971). In addition to receiving input from cortical regions, layer V of the entorhinal cortex receives output from the CA1 via the subiculum, which then projects to other brain regions, such as the mamillary nuclei, the thalamus, and the nucleus accumbens (Amaral, 1993; Wible, 2013).

Norman and O’Reilly (2003) developed a computational model of recognition memory that provides insight into the interplay between the hippocampal subregions, which function to mediate the storage and retrieval of recognition memory and may be transferable to other forms of hippocampal-dependent memories. During acquisition, incoming information is relayed from the neocortex to the hippocampus via the entorhinal cortex. From the entorhinal cortex, activation is spread both directly, and indirectly, via the dentate gyrus, to the CA3. The representations within the CA3 and dentate gyrus are very sparse, allowing for only a few units of information to be active for a given stimulus. This gives rise to pattern separation, based on the notion that, if only a few units are active per input pattern, there is decreased overlap between the hippocampal representations of different items (Norman and O’Reilly, 2003). Here, the dentate gyrus facilitates pattern separation in the CA3. From there, representations in the CA3 link to active units in the CA1, which also contains re-representation of the original input pattern. The CA1 serves as a relay station between the CA3 back to the entorhinal cortex, linking the sparse representations in the CA3 with the overlapping representations in the entorhinal cortex. When recalling a stored memory, strengthened connections in the entorhinal cortex-CA3 pathway, along with strengthened CA3 recurrents, allow for a reactivation of the CA3 pattern corresponding to the original item. From there, activation spreads to the representation in the CA1, and back to the representation in the entorhinal cortex. This representation loop allows the hippocampus to retrieve the complete version of the original input in response to a partial cue (Norman and O’Reilly, 2003).

## The CA2 Subregion of the Hippocampus

While the other subunits of the hippocampus have received extensive attention, the CA2 region has been largely neglected, poorly understood, and for the most part, unexplained (Chevaleyre and Siegelbaum, 2010; Caruana et al., 2012; Cui et al., 2013; Stevenson and Caldwell, 2014). Recent evidence has shed light on the obscurities of the CA2, characterizing it as a distinct subregion, with its own unique afferents and efferents, cellular composition, and contribution to hippocampal processes (Cui et al., 2013; Dudek et al., 2016; Mou, 2016).

Ramon y Cajal first described the hippocampus as a region with two distinct areas, which he divided based on the size of the pyramidal neurons that occupied the space: the *regio superior*, which was the top portion of the hippocampus and consisted of small pyramidal neurons, and the *regio inferior*, which was the lower portion of the hippocampus and consisted of large pyramidal neurons (Caruana et al., 2012; Dudek et al., 2016). Upon closer examination, Lorente de No (1934) reported that a small area within the *regio inferior* differed enough in its cytoarchitecture and connectivity to warrant a distinction from the rest of the area. From there, the *regio superior* became known as the CA1 and the *regio inferior* was divided into the CA2 and CA3 regions (Lorente de No, 1934; Dudek et al., 2016).

**FIGURE 5:**
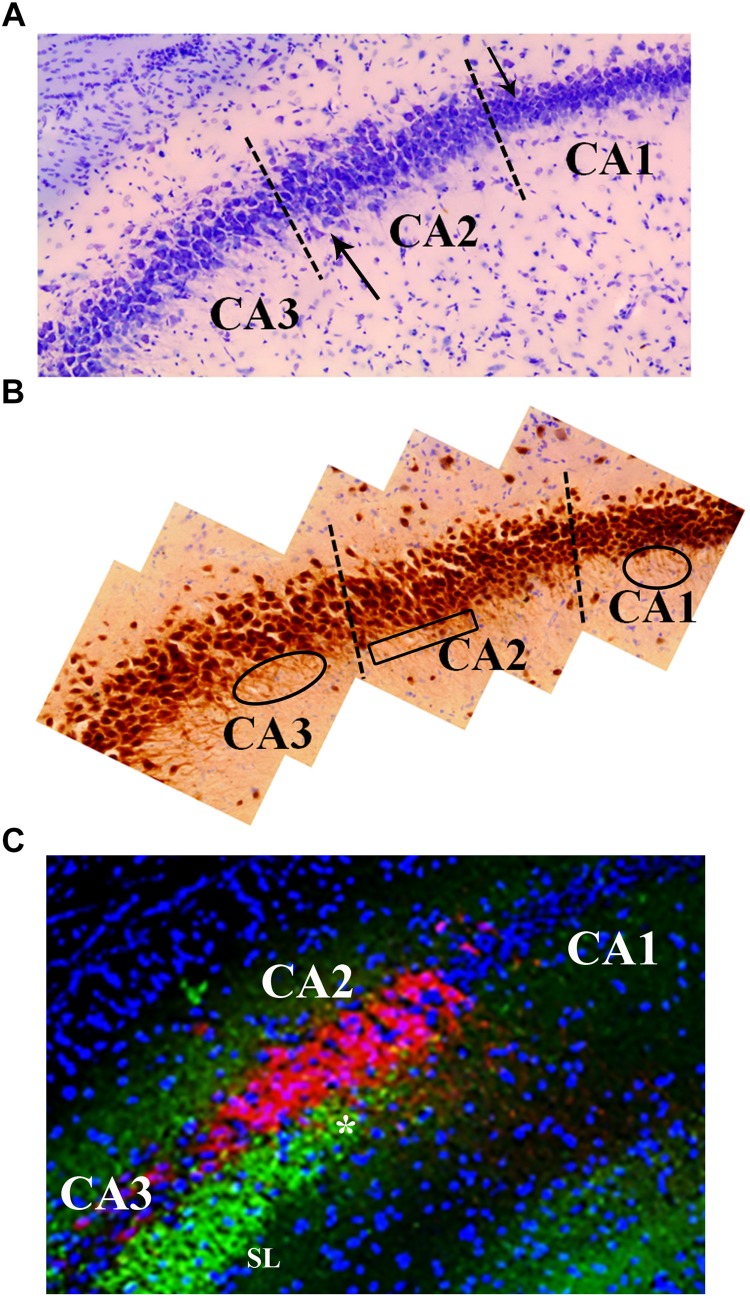
Neuroanatomical features that distinguish the CA2 region from the CA3 and CA1. **(A)** Cresyl violet staining shows subtle differences between the CA2 and CA3 regions. Namely, cresyl violet-stained cells in the CA2 region are smaller in size and more densely packed than the CA3 cells. There is also a slight widening of the cell layer at the border of CA2 and CA3 (upward pointing arrow). The higher density of cells in CA1 is a characteristic that delineates the CA2 from CA1 (downward pointing arrow). **(B)** The neuron specific stain, NeuN, exemplifies the orientation of neurons in the CA3, CA2, and CA1 regions. Specifically, the base of the apical dendrites from CA3 and CA1 pyramidal cells can be seen in this image indicating a coronal plane orientation (ovals). The base of the apical dendrites protruding from the CA2 neurons is less clear (boxed area) suggesting these dendrites are not in the coronal plane. The NeuN stain also highlights the difference in the width of the cell layers between CA2 and CA1 (visible at dashed line). **(C)** The CA2 region contains a high density of PEP-19 protein (Purkinje cell protein 4, pcp4; red in image). PEP-19 is a small calmodulin (CaM)-binding protein. This is a clear indication of the CA2 boundaries and distinguishes the CA2 region from the CA1 and CA3 where PEP-19 staining is absent. Also shown in this image is the *stratum lucidum* (SL; green: synaptophysin staining) where the mossy fibers terminate (highlighted by the high density of synaptophysin staining in green). The most dorsal aspect of the mossy fiber projection (asterisk) is adjacent to the CA2, PEP-19 staining pattern. Blue is DAPI staining showing cell layers.

The CA2 is situated between the CA3 and CA1 and is composed of a small population of large pyramidal neurons (Chevaleyre and Siegelbaum, 2010; Kohara et al., 2014). The cell bodies of the pyramidal neurons, like those of the CA3, are larger than those found in CA1 (Figures 5A,B). Unlike the CA3 pyramidal neurons, pyramidal neurons in the CA2 lack the specialized thorny excrescences on the apical dendrites associated with input from mossy fibers (Kohara et al., 2014; Dudek et al., 2016) yet receive mossy fiber input (Figure 5C). It was this morphological difference that prompted a separation of the *regio inferior* and was one of the first pieces of evidence that the CA2 was a distinct functional entity and not just an extension of the CA3 (Lorente de No, 1934; Amaral and Witter, 1989; Williamson and Spencer, 1994; Dudek et al., 2016; for additional differentiation, note PEP-19 staining in CA2 that is absent from CA3). In addition, unlike the dendrites of the CA3 pyramidal neurons, which extend along the longitudinal axis of the hippocampus, dendrites of CA2 neurons branch along the transverse axis of the hippocampus (Ishizuka et al., 1995; Chevaleyre and Siegelbaum, 2010; Figure 5B).

## CA2 Connectivity

**FIGURE 6:**
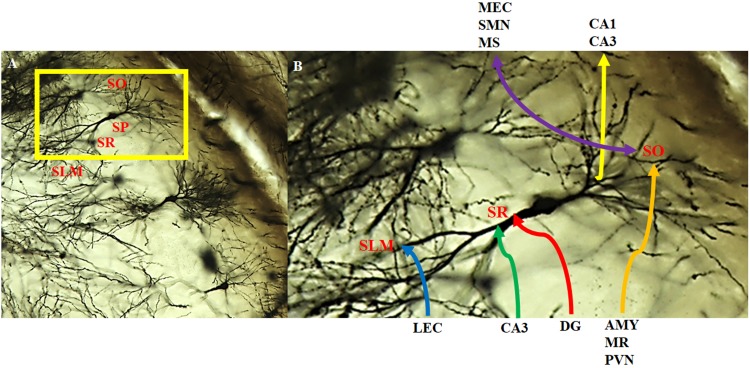
Intra- and extra-hippocampal connectivity patterns of CA2 neurons. Neurons within the CA2 region visualized with the Golgi-Cox method and magnified at **(A)** 20x and **(B)** 40x. CA2 neurons receive direct innervation from the lateral entorhinal cortex (LEC) via the temporoammonic pathway (blue). This input terminates in the distal dendritic field making up the *stratum lacunosum moleculare* (SLM). Intrahippocampal inputs arise from the CA3 (green) and the dentate gyrus (red; mossy fibers) that terminate on the more proximal dendritic field of the *stratum radiatum* (SR). CA2 neurons receive a substantial, extrahippocampal input (orange) from the amygdala (AMY), median raphe (MR), and paraventricular nucleus (PVN) that terminate on the basal dendritic field of the *stratum oriens* (SO). In addition, CA2 neurons make reciprocal, bilateral extrahippocampal connections (purple) with the medial entorhinal cortex (MEC), supramamillary nucleus (SMN) and medial/lateral septum/diagonal brand of Broca (MS). There are also feedback projections to CA3 pyramidal cells and feedforward to CA1 pyramidal cells (yellow). SP: *stratum pyramidale*.

The CA2 has been shown to receive both intrahippocampal and extrahippocampal inputs (Figure 6). While the CA2 is often excluded from hippocampal circuit diagrams, it fits within the traditional trisynaptic loop circuit (Caruana et al., 2012). Pyramidal cells within the *stratum lacunosum-moleculare* of the CA2 receive strong spatial input from cells in layers II and III of the medial entorhinal cortex, along with non-spatial information from layers II and III of the lateral entorhinal cortex (Figure 6B). The CA2 then projects strong unitary inputs to the *stratum oriens*, and to a lesser extent the *stratum radiatum*, in the CA1, synapsing onto basal dendritic arbors of CA1 pyramidal cells (Chevaleyre and Siegelbaum, 2010; Jones and McHugh, 2011; Caruana et al., 2012; Kohara et al., 2014; Mankin et al., 2015; Dudek et al., 2016; Figure 6B). This forms a unique indirect pathway of information transfer from the entorhinal cortex to the CA1 via the CA2 that parallels the direct pathway to CA1. This furthers the point that the CA2 is much more than a transition zone between CA1 and CA3, as was previously thought (Mankin et al., 2015). Pyramidal cells in the CA2 region have also been reported to project back to layer II of the medial entorhinal cortex (Rowland et al., 2013). The constant relay and flow of information to and from the entorhinal cortex has been known to render the quality of information susceptible to accumulating error. Coupled with the fact that, prior to the discovery of these direct, reciprocal CA2-entorhinal cortex inputs, the only known pathway between the entorhinal cortex and CA fields was a polysynaptic one, which could potentially lead to the loss of critical information necessary for recognition (McNaughton et al., 2006; Rowland et al., 2013). Given the importance of social recognition, particularly in terms of survival and social hierarchy, compromising any incoming information about an individual and their features could result in difficulties differentiating friend from foe. As such, this direct flow of information between the CA2 and entorhinal cortex could ostensibly ensure that little information is lost, allowing for a more viable memory trace and more accurate recognition. Reciprocal, bilateral connections also exist between the CA3 and CA2 regions, whereby the *stratum radiatum* of the CA2 receives weak, converging input from the CA3 via Schaeffer collaterals, and projects back to the *stratum oriens* of CA3 (Jones and McHugh, 2011; Caruana et al., 2012; Dudek et al., 2016; Figure 6B).

There is evidence that the mossy fiber terminals from the dentate gyrus may extend into the CA2 (Figure 6B) despite these neurons lacking dendritic thorny excrescences (Houser et al., 1990; Williamson and Spencer, 1994; Llorens-Martin et al., 2015). The absence of input from the dentate gyrus has been commonly used as an anatomical delineator between CA3 and CA2 regions. While this has been a common marker to distinguish the CA2 region from the CA3, it is not as reliable as the absence or presence of larger specialized post-synaptic markers (Dudek et al., 2016). Further investigations have also led to the understanding that the extent of dentate gyrus axon projection is species-specific (Dudek et al., 2016). The dentate gyrus has been reported to project axons to the *stratum radiatum* of the CA2 region in guinea pigs, cats, rats, and mice (McLardy, 1963; Hirama et al., 1997; Lavenex et al., 2009; Kohara et al., 2014; San Antonio et al., 2014; Dudek et al., 2016). Given the role of the dentate gyrus and its connectivity with the CA3 in spatial pattern separation, it is possible that the connections with CA2 mediate similar processes. Specifically, these connections may help the organism discriminate between a novel vs. familiar conspecific possessing similar features, especially considering that the dentate gyrus has been deemed necessary only when incoming information overlaps (i.e., may be similar) but has less of an impact when dissimilar information is processed (Lee and Solivan, 2010). Those same projections do not appear to occur in human and non-human primates (Lorente de No, 1934; Dudek et al., 2016). This may speak to the complexity of social recognition processing in human and non-human primate vs. other organisms; other organisms may rely on simple sensory input (smell, fur color, etc.) to discriminate between other individuals, which would potentially require simple circuitry to process the information. Social recognition in human and non-human primates is multi-factorial, involving the processing of emotion, context, etc., and not simply rely on the scent or singular physical feature of another to help recognize or discriminate between other organisms. As such, this processing would require more advanced circuitry and integration from other regions, and not rely solely on a dentate gyrus-CA2 connection to discriminate between all the different pieces of incoming information.

In addition to the intrahippocampal connections, the CA2 receives unilateral, extrahippocampal input from vasopressinergic cells in the paraventricular nucleus of the hypothalamus and the median raphe nucleus (Figure 6B). The CA2 also forms reciprocal, bilateral connections with the supramammillary nucleus, the medial and lateral septal nuclei, and the vertical and horizontal limbs of the diagonal band of Broca (Cui et al., 2013; Figure 6B). While the contribution of this innervation is not entirely clear, it has been hypothesized to modulate hippocampal excitability and play a role in memory consolidation (Pan and McNaughton, 2004; Shahidi et al., 2004; Caruana et al., 2012). Oxytocin and arginine vasopressin are important for modulating social behavior in a species-specific fashion (Donaldson and Young, 2008). They are synthesized in the paraventricular and supraoptic nuclei of the hypothalamus, with vasopressin also synthesized in the suprachiasmatic nucleus and released from the pituitary, where they bind to receptors found throughout the central nervous system and periphery (Gimpl and Fahrenholz, 2001; Insel, 2010). Furthermore, oxytocin and vasopressin have both been considered to play a role in learning and memory (Gimpl and Fahrenholz, 2001). As such, the connectivity between the CA2 and these extrahippocampal regions may mediate the neurochemical underpinning of social memory through the release and subsequent activity of these neuropeptides on the target brain regions which may explain the contribution of the hippocampus to social memory processing.

## LTP in CA2

Long-term potentiation (LTP) has been widely accepted to be the cellular underpinning of synaptic plasticity, and memory acquisition, storage, and retrieval in the central nervous system (Caruana et al., 2012). High-frequency stimulation of CA3 region, activating the Schaffer collateral inputs to the CA1, creates a robust potentiation within the CA1 region, inducing and sustaining LTP (Caruana et al., 2012). Similar induction of LTP is not observed within CA3-CA2 synapses. The inputs from CA3 via Schaeffer collaterals amass a very large feedforward inhibition onto the CA2 neurons (Chevaleyre and Siegelbaum, 2010; Jones and McHugh, 2011). Zhao et al. (2007) used whole cell recording to test whether 100 Hz synaptic stimulation could induce LTP in CA2 neurons. Upon stimulation, they reported no potentiation of the synaptic currents, specifically in the *stratum radiatum* of the CA2. They used a variety of methods, including the use of a perforated patch clamp technique, to try to induce LTP within the CA2, but to no avail (Zhao et al., 2007).

The high expression of TREK-1 and TREK-2 potassium channels in CA2 relative to other hippocampal subregions was a likely candidate for the lack of LTP, given that these two-pore channels create a potassium-mediated leak current, significantly hyperpolarizing the resting membrane potential and requiring a greater depolarizing current to initiate LTP (Zhao et al., 2007; Caruana et al., 2012). Blocking potassium channels, including TREKs, with cesium in the electrode internal solution still inhibited LTP induction (Zhao et al., 2007; Dudek et al., 2016). LTP inhibition was linked to an abundance of STEP, a phosphatase that can dephosphorylate and inactivate ERK in response to NMDA receptor activation and can inhibit NMDA function (Zhao et al., 2007; Caruana et al., 2012). Application of okadaic acid, a protein phosphatase inhibitor, failed to induce LTP (Zhao et al., 2007). Induction of LTP was still absent when GABA_*A*_ inhibitory currents were blocked using picrotoxin, yet all of these protocols consistently induced LTP in the CA1 (Zhao et al., 2007; Caruana et al., 2012).

Given that certain forms of LTP in the hippocampus are mediated by NMDA receptors, Zhao et al. (2007) compared the patterns of expression of NMDA receptor mRNA in the CA2 and CA3 and found that they were expressed evenly across both regions. They concluded that critical components of the LTP were missing in the CA2 and offered some molecular causes to explain the lack of LTP (Zhao et al., 2007). One possible explanation is the abundance of A_1_ adenosine receptors, of which the population is denser in the CA2 region than other hippocampal regions (Zhao et al., 2007; Jones and McHugh, 2011). Adenosine is a well-known potent inhibitory modulator. It is a byproduct of both intracellular and extracellular ATP metabolism and is released with other neurotransmitters and metabolized by ectoprotein kinases (Wieraszko et al., 1989; Mitchell et al., 1993). Stimulation of adenosine A_1_ receptors activates potassium channels and inhibits calcium channels, both of which result in a hyperpolarization of hippocampal neurons. The resulting hyperpolarization and reduction in calcium influx counteracts the post-synaptic depolarization that is required for LTP and suggests that the lack of LTP in the CA2 may be mediated by adenosine (Arai et al., 1990; de Mendonca and Ribeiro, 1990; Zhao et al., 2007).

Another explanation offered by Zhao et al. (2007) was that CA2 pyramidal neurons expressed calbindin, a calcium-binding protein found in inhibitory interneurons that can regulate synaptic plasticity. Since calcium signaling is important for the induction of LTP in other regions of the hippocampus, it is possible that the inhibition of LTP may reflect differences in local calcium handling in CA2 (Simons et al., 2009; Caruana et al., 2012). To test this hypothesis, Simons et al. (2009) loaded CA2 pyramidal neurons with a fluorescent calcium dye indicator and used two-photon confocal imaging to study the dendrites and spines of those neurons. They reported significantly smaller evoked calcium transients in CA2 compared to those in CA1 and CA3 (Simons et al., 2009). From there, they calculated a significantly higher endogenous calcium buffering capacity, along with significantly higher calcium extrusions, in the CA2 relative to CA1 and CA3. After raising the extracellular calcium levels of the preparation and using a higher frequency of tetanic stimulation, they managed to induce LTP at CA2 *stratum radiatum* synapses (Simons et al., 2009; Caruana et al., 2012; Dudek et al., 2016). They concluded that these observations could account for the lack of LTP propagation in the CA2 (Simons et al., 2009). This finding indicated that, while the CA2 contains a slew of proteins that may contribute to the failure of LTP induction, these synapses nonetheless possess the intracellular machinery necessary for LTP induction, but that higher calcium buffering and extrusion prevents plasticity (Simons et al., 2009).

The CA2 region possesses certain ‘memory inhibiting’ genes, of which one is RGS14. RGS14 is highly enriched in CA2, specifically in the *stratum lacunosum-moleculare* and *stratum radiatum* and has been shown to play a crucial role in the suppression of LTP induction (Lee et al., 2010; Dudek et al., 2016). It is a scaffolding protein that integrates G protein and H-Ras/ERK/MAP kinase signaling pathways (Lee et al., 2010). Lee et al. (2010) created a transgenic mouse line, which lacked the RGS14. They reported a robust capacity for LTP within CA2 pyramidal neurons in the transgenic mice. They then evaluated the effect of RGS14 silencing on hippocampal-based spatial learning and memory. They reported that silencing of RGS14 resulted in marked improvements in both the Morris water maze and novel-object recognition task. They suggested that RGS14 my act as a natural brake to limit LTP and synaptic plasticity within the CA2 following stimulation. This may result from RGS14 binding to various kinases, inhibiting growth-factor directed MAP kinase signaling (Lee et al., 2010). They concluded that RGS14 regulates signaling pathways linking synaptic plasticity in CA2 to hippocampal-dependent memory processes, distinct from the trisynaptic pathway (Lee et al., 2010).

Given this lack of demonstrable LTP, it may be tempting to presume that the CA2 is a highly stable component of the hippocampal circuit, devoid of any plastic functions common to the hippocampus. However, investigations have provided evidence suggesting that the CA2 is, in fact, highly plastic, though highly selective for the type of stimulus that can induce LTP (Caruana et al., 2012). The abundance of adenosine vasopressin (AVPR) 1b and adenosine A_1_ receptors have been shown to have powerful and selective effects on synapse within the CA2 region, inducing a long-lasting potentiation of synaptic transmission within the area following the release of vasopressin or oxytocin (Caruana et al., 2012). As such, the induction of LTP, and concurrent memory formation, within the CA2 occurs under very specific conditions, e.g., vasopressin and oxytocin receptor activation, which happen to be neuropeptides that highly regulate social memory and behaviors. This provides further support that LTP and synaptic plasticity at CA2 synapses play a role in social recognition, and provides further insight into the function of the aforementioned connectivity between the CA2 and extrahippocampal regions (Wersinger et al., 2002; Prediger and Takahashi, 2005; DeVito et al., 2009; Caruana et al., 2012).

## CA2 Function

Though apparently resistant to plasticity, evidence exists to suggest that the CA2 plays an integral role in various memory processes. DeVito et al. (2009) assessed memory processes in AVPR1b-knockout mice. AVPR1b has been shown to play an important role in regulating social behaviors, but their role in hippocampal-dependent memory function has yet to be investigated. The highest level of expression of AVPR1b in the brain is in the CA2 subregion (Wersinger et al., 2002; Young et al., 2006; DeVito et al., 2009). DeVito et al. (2009) employed an object recognition task and found that the knockout mice spent the same amount of time exploring a recently observed stationary object as they did an older one. These results suggest an impairment in memories that involve a temporal component. They further concluded that the involvement of CA2, in particular AVPR1b within the CA2, might play a highly selective role in temporal processing that supports episodic memory (DeVito et al., 2009).

Further investigations into the contributions the CA2 makes in memory processes lead investigators to uncover a potential role in contextual learning. Wintzer et al. (2014) used compartment analysis of temporal activity fluorescence *in situ* hybridization (catFISH) imaging to compare cell ensembles in CA1, CA2, and CA3 subregions in mutant mouse lines lacking either NMDA receptors at the CA3 synapse (CA3-NR1 KO) or at the perforant path inputs from the entorhinal cortex to the dentate gyrus (DG-NR1 KO). This allowed the investigators to manipulate the quality of incoming information to CA2. CA3-NR1 KO leads to deficits in rapid encoding information and associative memory storage, making a familiar representation more novel, while DG-NR1 KO leads to deficits in context discrimination, making a novel representation more familiar (Nakazawa et al., 2002; McHugh et al., 2007; McHugh and Tonegawa, 2009; Wintzer et al., 2014). The mice were subjected to familiar, altered, and novel context environments. The investigators reported that cell ensembles in CA2 were particularly sensitive to subtle changes in familiar environments; that is, they observed a robust change in ensembles in the CA2 that were disproportionate to the magnitude of contextual change (Wintzer et al., 2014; Dudek et al., 2016). This results from a remapping in CA2, independent of what is observed in either CA3 or CA1, uncoupling the CA2 from both regions (Wintzer et al., 2014). They concluded that the CA2 functions to detect and encode changes in patterns of input, in effect detecting conflicts between what the hippocampus recalls and what the animal is experiencing. One of the attributes to this function may result from the extrahippocampal input the CA2 receives from the supramammillary nucleus, a region activated by novelty (Wintzer et al., 2014).

Mankin et al. (2015) recently investigated the role CA2 plays in processing spatial aspects of memories. They analyzed spatial firing patterns in rats that randomly foraged in highly familiar environments in the morning and, 6 h later, in the afternoon. A higher mean spatial firing rate without a difference in peak firing rate among all spatial locations was reported in the CA2 relative to the other regions. This suggests that the amount of spatial information per cell in the CA2 was lower compared to the other regions (Mankin et al., 2015). The CA2 was also reported to possess a higher number of place fields per cell, as well as a larger field size, compared to the other subregions. Mankin et al. (2015) suggested that the combination of a larger number of place fields per cell and an increase in field size accounted for the reduction of spatial information. Spatial firing patterns in the CA2 were as consistent as in the CA3 or CA1 when the rats visited the same box shape repeatedly. These same firing patterns in CA2 became less consistent for repeated sessions in the same box shape that were separated by an intervening session of another shape. These results indicate that place fields in the CA2 do not show a persistent code for space or for changes in context, but rather become increasingly dissimilar in response to the passage of time, suggesting a role in temporal coding. These results indicate that, while the CA2 plays some role in spatial processing, it is not equivalent to that played by the CA3 and CA1 (Mankin et al., 2015; Dudek et al., 2016).

According to Mankin et al. (2015), the unique inputs from CA2 converge with other inputs to CA1, differing the CA1 activity patterns from one time to another. This convergence of time-varying inputs would create a time-stamped neural code that differs between similar events at different times. In addition, there would be greater overlap within the neural code for events that occurred closer in time (Mankin et al., 2015). Therefore, while observing the CA2 at the individual cellular level may demonstrate properties consistent with a role in spatial coding, the population level points to more of a role in temporal coding and less of a role in spatial contextual coding (Mankin et al., 2015; Dudek et al., 2016). Contrary to the findings reported by Wintzer et al. (2014), which suggest that CA2 plays a crucial role in contextual coding, Mankin et al. (2015) reported that the CA2 showed weak discrimination between spatial contexts; that is, the place fields in this region showed less change in response to alterations in the shape of a context compared to other regions. As Dudek et al. (2016) pointed out in their review, both studies differed in how the context was changed. They noted that, while Wintzer et al. (2014) introduced a novel change to a familiar environment, the experimental protocol employed by Mankin et al. (2015) used a familiarized change in the shape of the box (Dudek et al., 2016). Taken together, results from both studies suggest that the CA2 is required when a context is being updated, in addition to temporal coding (Dudek et al., 2016).

The CA2 inputs into both CA3 and CA1 place the CA2 region in a good position to influence network physiology and information processing within the hippocampus (Boehringer et al., 2017). In order to assess the role of CA2 in hippocampal network function, Boehringer et al. (2017) silenced synaptic transmission of CA2 pyramidal cells both chronically, via the expression of the tetanus neurotoxin light chain, and acutely, via the activation of the G_i_-coupled DREADD receptors. They reported that chronically silencing the CA2 lead to a hyperexcitability within the recurrent CA3 circuit during exploration. In addition, acute silencing caused “hotspots” of multi-unit activity resulting from an increase in the concentration of CA1 and CA3 pyramidal cell spiking (Boehringer et al., 2017). They then examined the chronic loss of CA2 output and its effect on hippocampal activity during periods of immobility. Chronic loss of CA2 output lead to the appearance of large-amplitude, short-duration epileptiform-like discharges, which were initially observed in CA3 and then CA1, indicating they were CA3-driven. They suggested that CA2 silencing lead to a replacement of normal ripples with epileptiform-like discharges due to abnormally strong CA3 input arising in CA2 (Boehringer et al., 2017). To assess acute CA2 silencing on memory processes, Boehringer et al. (2017) habituated mice to a single context, followed by a distinct novel context. They reported slower habituation in CA2-silenced mice, indicating that the CA2 is integral in contextual habituation (Boehringer et al., 2017). Taken together, these results suggest that CA2 acts as a regulator of network processing within the hippocampus, creating a balance between excitatory and inhibitory processes required for proper network function, and preventing the hippocampus from entering a state of pathological activity. In effect, the CA2 functions to prevent “overheating” of the entire hippocampal network (Boehringer et al., 2017).

Based on the presented investigations, the evidence supports the hypothesis that the CA2 plays in integral role in the temporal and contextual processing of information. In terms of social memory, one can deduce that the CA2 plays a role in delineating whether a conspecific was encountered recently or remotely, which may facilitate the recognition of another organism, especially one that was encountered recently. Additionally, the CA2 acts as a sort of hippocampal error detector, allowing for the differentiation between a novel context and one that is familiar but with slight variation. This can prove to be extremely useful in terms of social memory as it allows an organism to constantly adapt to changes within their surroundings, and those who surround them. Without this ability, any detectable change in the recognition cue of a familiar conspecific (scent, appearance, etc.) or the environment in which the conspecific was initially encountered could ostensibly create errors in recognition. Together with the temporal processing of information, the CA2 can be viewed as processing the general ‘when, where, and what’ aspects of social recognition.

## CA2 Pathology

Investigations into the distinct physiology and unique cognitive contributions of CA2 have cultivated some novel insight into the role this region plays in a myriad of neuropsychological disorders. A postmortem study completed by Benes et al. (1998) was one of the first to point out the contribution of CA2 to neuropsychiatric disorders. In their investigation, there was a 40% reduction in the number and density of non-pyramidal neurons in the *stratum pyramidale* of CA2 in schizophrenic and manic-depressive patients compared to controls (Benes et al., 1998). Given that both schizophrenia and manic-depressive disorders present with different clinical presentations and unique pathophysiologies, there is unlikely to be a strictly genetic explanation for the reduction in non-pyramidal neurons observed, but perhaps an environmental one. Stress would seem to be a likely contributor to the non-pyramidal cell reduction, given that all forms of stress are associated with in an increased release of glucocorticoids, which has been shown to have a particular affinity for the CA2 region and may potentially exert a more robust effect on neurons in this region (Benes et al., 1998). Glucocorticoids have been found to increase excitotoxicity in hippocampal neurons, which could have contributed to the decrease of non-pyramidal neurons in the CA2 (Benes et al., 1998).

Zhang and Reynolds (2002) reported a significant deficit in the density of parvalbumin-immunoreactive neurons in the CA2 of patients with schizophrenia. These GABAergic interneurons are directly involved in the inhibitory control of pyramidal cell output activity, and as such, a deficit may result in increased activity of excitatory pyramidal neurons (Zhang and Reynolds, 2002). These findings may point to neurodevelopmental etiology for schizophrenia, based on the observation that parvalbumin expression occurs substantially later than other calcium-binding proteins in the hippocampus. This creates a period of vulnerability, whereby immature GABAergic cells may be particularly susceptible to neurotoxic events before the expression of parvalbumin can serve as protection (Zhang and Reynolds, 2002). Precisely how CA2 dysfunction contributes to positive, negative, or cognitive symptoms remains unclear, although the cognitive decline may be associated with a disruption in filtering of mnemonic information in the hippocampus (Jones and McHugh, 2011).

While both CA3 and CA1 pyramidal cells are extremely vulnerable to damage caused by epilepsy, CA2 pyramidal neurons are mostly spared (Sperk, 1994). After employing sustained electrical stimulation of the perforant path in rats, Sloviter (1983) observed large amplitude population spikes and epileptiform discharges from hippocampal granule cells, leading to a morphological damage in CA3 and CA1 pyramidal cells, while CA2 pyramidal cells were relatively unaffected. Based on these results, Sloviter (1983) hypothesized that the damage to CA3 pyramidal cells resulted from excessive excitation by granule cells in the dentate gyrus, while the damage to CA1 pyramidal cells was due to their sustained activation by the perforant path. The sparing of CA2 pyramidal cells seen in epileptic brains may result from their lack of dendritic thorns connecting with mossy fiber input from the granule cells, which means they would not be subjected to the same excitotoxic damage as would CA3 cells (Sloviter, 1983). Similarly, Williamson and Spencer (1994) reported that CA2, but not CA3 or CA1, cells appear to be resistant to damage caused by sustained epileptiform bursts found in patients with medial temporal lobe sclerosis, a common neuropathology found in temporal lobe epilepsy. They hypothesized that the calcium-binding proteins parvalbumin and calbindin-128D provide some form of neuroprotection against excitotoxic damage, given that the CA2 pyramidal cell possesses higher levels of these proteins than other populations of cells that are lost in medial temporal lobe sclerosis (Williamson and Spencer, 1994).

## CA2 and Social Recognition

Investigations have implicated the CA2 as being a subregion paramount to social memory processes. Recent reports into the cellular components of the CA2 have provided further evidence for its role in social recognition. Young et al. (2006) performed *in situ* hybridization in mice brains and reported that the labeling of AVPR1b was the highest in the dorsal CA2 pyramidal cells and confirmed these findings with reverse transcriptase-polymerase chain reaction. They speculated that the CA2 field plays a role in the formation or retrieval of memories of social encounters, much like how other hippocampal pyramidal cells establish memories of place (Young et al., 2006). Expanding on this, Smith et al. (2016) used optogenetics to excite vassopressin terminals, originating from the paraventricular nucleus of the hypothalamus, in the CA2 to explore the involvement of AVPR1b-expressing neurons in the CA2. They observed enhanced social memory when optical stimulation occurred during memory acquisition, but not during retrieval. They then administered an AVPR1b antagonist directly into the CA2 and reported an impairment in the enhancement of social memory (Smith et al., 2016). These results suggest that direct stimulation of vasopressin terminals that innervate CA2 enhances memory formation and extends social memories (Smith et al., 2016). In addition, this hypothalamic-CA2 pathway, along with its role in social recognition is reliant on AVPR1b signaling (Young et al., 2006).

The speculation that the CA2 region may integrate olfactory cues with social encounters in order to create an appropriate response becomes apparent when considering the direct inputs CA2 receives. For one, the CA2 receives non-spatial information from the entorhinal cortex (Chevaleyre and Siegelbaum, 2010; Jones and McHugh, 2011; Caruana et al., 2012; Kohara et al., 2014; Mankin et al., 2015; Dudek et al., 2016). The entorhinal cortex receives input from the olfactory bulb and piriform cortex, both areas that process olfactory information (Boeijinga and Van Groen, 1984). In addition, the CA2 is the only region within the hippocampus to receive vasopressinergic inputs from the paraventricular nucleus of the hypothalamus, which appear crucial to mediating social behavior (Cui et al., 2013; Zhang and Hernandez, 2013). Thus, it is possible that the CA2 incorporates both forms of input, e.g., social and olfactory, to create a memory trace that is reliant on the scent of the individual.

Recent advancements in genetic manipulation techniques have allowed for investigations into the role of CA2 in social cognition in a more precise and sensitive manner. Hitti and Siegelbaum (2014) used a Cre-expressing mouse line, known as *Amigo2-Cre*, to inactivate pyramidal neuron output from the CA2. They injected a Cre-dependent AAV into the dorsal hippocampus to express tetanus neurotoxin (TeNT) light chain in CA2 pyramidal neurons, which blocked neurotransmission. To test the efficacy of this method, they co-expressed channelrhodopsin-2 (ChR2) with either TeNT or yellow fluorescent protein (YFP) using Cre-dependent AAVs. From there, they used whole-cell current clamp recordings to determine the strength of synaptic transmission from CA2 to CA1 pyramidal neurons. Focal photostimulation over a wide range of intensities delivered to CA1 *stratum oriens* and *stratum radiatum* regions evoked robust monosynaptic potentials in the CA1 pyramidal neurons in slices in which ChR2 was co-expressed with YFP in CA2 pyramidal neurons. Slices in which ChR2 was co-expressed with TeNT in CA2 pyramidal neurons produced little to no synaptic response in CA1 neurons when CA2 neurons were illuminated (Hitti and Siegelbaum, 2014).

Given the efficacy of TeNT lesions, they then investigated the behavioral consequences of CA2 inactivation. A three-chamber social novelty test was used to compare performance between control mice, expressing YFP in CA2 pyramidal neurons, and CA2-TeNT mice. In this test, the amount of time that the experimental mouse spent interacting with a novel, unrelated mouse was compared to the time spent interacting with a familiar co-housed littermate in order to measure social recognition (Hitti and Siegelbaum, 2014). While the CA2-YFP control group showed a significant preference for the compartment containing the novel animal, the CA2-TeNT group did not. Additionally, the difference between the time spent exploring the novel mouse and the time spent exploring the familiar mouse was significantly less in CA2-TeNT mice compared to CA2-YFP mice (Hitti and Siegelbaum, 2014). Since neither a defined learning phase nor a delay period was incorporated in this social novelty test, a direct interaction test was used to measure social memory (Hitti and Siegelbaum, 2014). The direct interaction test exposed the experimental mouse to an unfamiliar one in trial 1, and, after a 1-h inter-trial interval, either re-exposed the experimental mouse to the same mouse that it encountered in trial 1 or exposed it to a second, unfamiliar mouse (Hitti and Siegelbaum, 2014). The CA2-TeNT group demonstrated impaired social memory, given that there was no decrease in time that the CA2-TeNT mouse spent exploring a previously encountered mouse (Hitti and Siegelbaum, 2014).

A five-trial habituation/dishabituation social memory was used to confirm that CA2-inactivation resulted in impaired social memory. In this test, the experimental mouse was exposed to a stimulus mouse for four consecutive trials then introduced to a novel stimulus mouse on the fifth trial. The CA2-TeNT mice showed no significant habituation during the first four trials as evidenced by a lack of decreased exploration and no significant dishabituation to the novel mouse as shown by a lack of increased exploration (Hitti and Siegelbaum, 2014). The CA2-YFP mice showed both a marked habituation and dishabituation during the test, which confirmed the necessity of the CA2 for social memory (Hitti and Siegelbaum, 2014).

A three-chamber test of sociability was used to compare performance between CA2-YFP mice and CA2-TeNT mice. This test examined the normal preference of the experimental mouse for a chamber containing a littermate versus an empty chamber. Both the CA2-YFP and CA2-TeNT groups displayed a significant preference for the compartment containing their littermate, suggesting that CA2 inactivation did not alter sociability (Hitti and Siegelbaum, 2014). In their social novelty test, Hitti and Siegelbaum (2014) also reported that CA2-YFP and CA2-TeNT mice showed similar and unchanging exploration times during trials 1 and 2 when two novel mice were encountered in the two trials indicating that the sociability of the CA2-TeNT mice remained intact. Furthermore, CA2 silencing did not impair other hippocampus-dependent memory process, including novel-object-recognition and spatial memory, nor did it alter locomotor activity, anxiety-like behavior, or olfaction (Hitti and Siegelbaum, 2014). Thus, the impairment of the CA2 region and subsequent ablation of social recognition provides evidence for the crucial role the CA2 has in social cognition (Hitti and Siegelbaum, 2014).

Following up to the work by Hitti and Siegelbaum (2014) and Stevenson and Caldwell (2014) performed bilateral excitotoxic NMDA lesions of the CA2 region in mice to determine the effects on social recognition memory. To accomplish this, they employed two social memory test following surgical lesions of the CA2. In the first test, the experimental mouse was exposed to a stimulus mouse for 5 min, which was then removed, and reintroduced for the second trial 30 min later, along with a novel mouse. Control mice spent significantly more time investigating the stimulus mouse in the first trial than CA2-lesioned animals. In the second trial, the control mice spent significantly more time with the novel mouse than the familiar one, while CA2-lesioned rats demonstrated no difference in the amount of time spent investigating either mouse (Stevenson and Caldwell, 2014). In the second test, the experimental animal was exposed to a stimulus mouse for 1 min, which was then removed for 5 min; this series of exposures with same familiar mouse occurred for a total of 10 trials with an inter-trial interval of 5 min. On the 11th trial, the experimental mouse was then exposed to a novel mouse. The CA2-lesioned mice spent significantly less time with the stimulus mouse presented throughout the 10 trials, as well as significantly less time investigating the novel mouse on the 11th trial, compared to control mice (Stevenson and Caldwell, 2014). Together, the results from both studies suggest an impairment in social recognition in CA2-lesioned mice. Stevenson and Caldwell (2014) went on to discuss the parallels between their results and the investigations into AVPR1b-knockout mice; social recognition deficits were noted in both cases. They suggested that AVPR1b within the CA2 may be important to display specific social behaviors, in particular the coupling of olfactory information with the “what” components of odor context. Therefore, inactivation of the CA2 would ablate the AVPR1b-driven associations between both components (Stevenson and Caldwell, 2014).

## Conclusion

The CA2 is a dynamic region with a composition and connectivity unique to the hippocampus, and not just an extension of the CA3, as was previously believed. Given that research into this region is a relatively new endeavor, the precise role of the CA2 is not fully understood. Nevertheless, burgeoning research into this area has established the role of CA2 in social memory processes, with a particular focus on social recognition, and as such, dysfunction in this area has been implicated in the social impairments common to a number of psychiatric diseases. Beyond these preliminary findings, little is known concerning the cellular pathways that regulate this brain region and how it mediates social recognition representations, while its contribution to neuropathologies is somewhat vague and inferential, at best. Further research into the cellular mechanisms that govern the CA2 may provide insight into the social deficits underlying certain neuropathologies, such as autism and schizophrenia, and potentially yield novel treatment strategies for these disorders.

## Author Contributions

NT wrote the initial and revised versions of the manuscript. MH commented and edited the manuscript. Both authors approved the final manuscript for submission.

## Conflict of Interest

The authors declare that the research was conducted in the absence of any commercial or financial relationships that could be construed as a potential conflict of interest.
